# LINDA – a solution-focused low-intensity intervention aimed at improving health behaviors of young females: a cluster-randomized controlled trial

**DOI:** 10.1186/1471-2458-13-1044

**Published:** 2013-11-04

**Authors:** Päivi Valve, Susanna Lehtinen-Jacks, Tiina Eriksson, Matti Lehtinen, Pirjo Lindfors, Marja-Terttu Saha, Arja Rimpelä, Susanna Anglé

**Affiliations:** 1 School of Medicine, University of Tampere, 33014 Tampere, Finland; 2 Pirkanmaa Hospital District, Science Centre, 33521 Tampere, Finland; 3 School of Health Sciences, University of Tampere, 33014 Tampere, Finland; 4 Nutrition Unit, National Institute of Health and Welfare, P.O. Box 30, 00271 Helsinki, Finland; 5 Department of Paediatrics, Tampere University Hospital, 33521 Tampere, Finland; 6 School of Medicine, Department of Paediatrics, University of Tampere, 33521 Tampere, Finland; 7 Department of Adolescent Psychiatry, Tampere University Hospital, 33380 Pitkäniemi, Finland

**Keywords:** RCT, Solution-focused, Physical activity, Meal pattern, Sleep, Overweight, BMI

## Abstract

**Background:**

We aimed to develop and evaluate the effectiveness of an individualized, long-term support lifestyle counseling approach in promoting healthy physical activity, improving dietary and sleeping behaviors, and preventing weight gain in young females. The counseling approach’s intensity was designed to be low enough to be implementable in primary health care.

**Methods:**

Young women (n = 3,059, age at baseline 17–21 years) attending a population-based human papilloma virus vaccination trial (clinicaltrials.gov identifier: NCT00122681) in 15 vaccination centers in different communities across Finland, were cluster-randomized into intervention and control arms of the LINDA intervention. Both intervention and control arms received counseling on sexual health and contraception from the study nurses as part of the vaccination trial. Additionally, the LINDA intervention arm (n = 1,537) received a 20-minute individualized lifestyle counseling session followed by further support at the six-monthly follow-up visits of the vaccination trial, in total for 1.5–2.5 years.

The LINDA solution-focused brief therapy intervention focused on healthy physical activity, and dietary and sleeping behaviors, based on the needs and interests of the participants. Anthropometrics were measured, and data on health-related behaviors were collected using self-report questionnaires at baseline and after the intervention at 1.5–2.5 years.

**Results:**

In the intervention arm, 37% vs. 31% in the control arm made an overall improvement in their health behaviors concerning physical activity, meal regularity and/or earlier bedtime (NNT = 18, 95% CI = 11–50). The per-protocol analysis further revealed that 30% of those who actually received lifestyle change support on healthy physical activity behaviors improved their physical activity level vs. 23% in the control group (NNT = 15, 95% CI = 9–38). Respectively, 36% of those who received support on healthy sleeping behaviors went to sleep earlier before school-/work-days after the intervention vs. 28% in the control group (NNT = 13, 95% CI = 7–61). Dinner irregularity increased in both groups, but less in the intervention group among those who received support on healthy dietary behaviors (NNT = 15, 95%CI = 9–46). There was no effect on weight gain between baseline and study end-point.

**Conclusions:**

The solution-focused brief therapy intervention, with individually tailored content, helped to make small, long-term overall improvements in health behaviors concerning physical activity, meal regularity and/or earlier bedtime.

## Background

Physical activity, diet, sleep and weight status are major determinants of health and well-being. The prevalence of overweight and obesity has increased in many parts of the world during the last decades [1,2]. The percentage of overweight or obese 12–18-year-old Finnish adolescents tripled from 1977 to 2005 [3,4], and in 2010, 14% of 15–24-year-old females were overweight [5]. Since girls often underestimate their weight [6], it is possible that the above figures which are based on self-reported height/weight are actually higher. Lack of physical activity among adolescent Finns is alarming: only 30% of 16–18-year-olds were physically active enough and 30–35% were physically inactive [7,8].

An increasingly sedentary lifestyle and unhealthy diet result in an imbalance between energy intake and expenditure [9]. Sleep contributes to both sides of the energy balance equation, and inadequate sleep has been suggested to be a risk factor for obesity [10,11].

Because permanent weight reduction is difficult, the easiest approach to weight control is the prevention of weight gain. In Finland, promoting healthy nutrition and physical activity is common, and includes both society-oriented actions and actions targeting individuals [12]. For example, the national type 2 diabetes prevention program 2003–2010 [13] addressed nutritional and physical activity education, and obesity prevention of the entire population, including young adults. This was done, for example, by simple lifestyle counseling in health examinations. For young adults, there is, however, typically a health examination only once at the beginning of university, vocational studies or employment [13].

At least three 1-year interventions involving individualized counseling have previously targeted improvement of lifestyle habits and/or prevention of weight gain in adolescents [14-16] or young women [17].

The PACE + study [14], which was aimed at improved nutrition and physical activity among US adolescents (healthy 11–15-year-olds, recruited from primary care), consisted of a computer-assisted diet and physical activity assessment, an according stage-based goal setting, a brief (3–5 min) counseling session with a health care provider, and monthly tailored counseling by mail and telephone (10–15 minutes per session) as well as parental involvement. Statistically significant improvements between the groups occurred only in the reduction of sedentary behavior (screen time), but not in physical activity, dietary indicators or BMI z-scores [14].

The New Moves obesity prevention program was aimed at US adolescent girls (grades 9–12 at school), who were overweight or at risk of becoming overweight due to low levels of physical activity [15,16]. The original program, comprising physical activity, nutrition and social support sessions at school, improved only one (stage of change for physical activity) of the several outcomes related to physical activity, dietary habits and BMI. Importantly, adding individual counseling sessions using motivational interviewing to the program [16] led to improvements (as compared with the control group) in several outcomes concerning sedentary activity, physical activity, dietary intake, eating patterns, unhealthy weight-control behaviors, and body-/self-image, although not in body composition or BMI.

The Health Hunters study [17], which aimed at preventing overweight and obesity, targeted 18–28-year-old Swedish women with at least one severely obese parent. The intervention consisted of a face-to-face examination and counseling session, followed by regular personalized contact via telephone and email, as well as occasional group sessions. The staff supported the subjects in developing individual strategies for overcoming barriers to more healthy lifestyles. BMI decreased in the intervention group significantly while it increased in the control group. There was also a significant increase in self-reported physical activity in the intervention group compared with the control group, but there was no effect on any of the dietary indicators [17].

The purpose of the present study was to find out whether individualized solution-focused counseling with a long maintenance support time would bring an additional benefit compared with the standard care in Finland, as regards promoting healthy physical activity, dietary and sleeping behaviors, and preventing weight gain.

## Methods

### Study population

**Figure 1:**
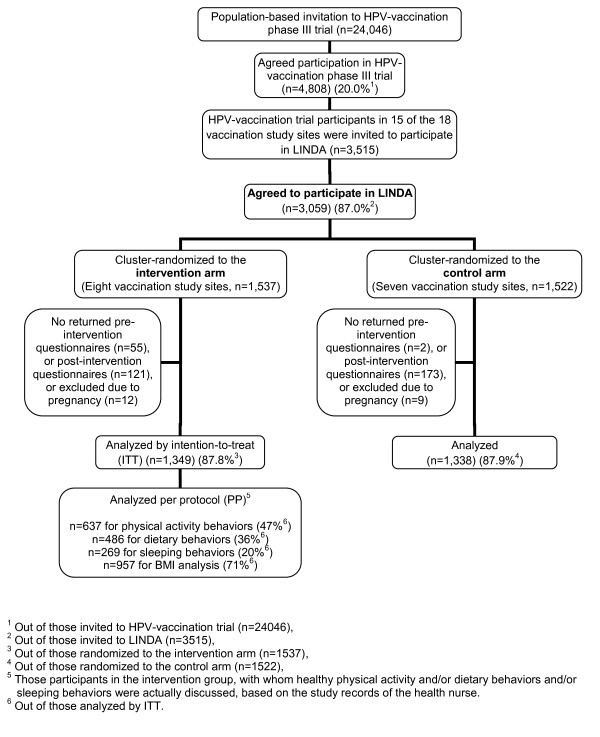
Participant enrollment, randomization, and adherence to the study.

The participants were recruited from the population-based human papilloma virus (HPV) vaccination trial, HPV-008 [18], which was organized in fifteen vaccination centers in different communities across Finland in 2006. The participants were Finnish females aged 17–21 at the baseline of the present study. Of the invitees, 87% agreed to take part in the LINDA low-intensity intervention, as shown in Figure 1. Pregnancy was an exclusion criterion.

### Study design

The study was designed as a cluster-randomized controlled trial, as a sub-study of the HPV-008 vaccination trial [18]. The participants were cluster-randomized into intervention and control groups based on the vaccination center (community), as described in [19]. The randomization was done by exploiting random numbers assigned to the vaccination centers (communities) stratified by median community size. After randomization, the intervention group contained participants from eight vaccination centers, and the control group from the other seven vaccination centers. The girls in both groups received counseling on sexual health as a part of the HPV vaccination study. In addition, the LINDA intervention group received lifestyle counseling from the study nurses during their visits for the HPV study. In the control group, the girls followed the HPV-008 study protocol [18], including lifestyle counseling according to standard care in Finland.

### Description of the LINDA intervention

LINDA is an acronym of a Finnish expression “brief intervention for ladies” (LyhytINterventio DAameille). The LINDA intervention comprised components deemed effective in a recent systematic review of dietary and physical activity interventions [20]. Since social support is important and the use of behavior-changing techniques (e.g. goal-setting, self-monitoring) is essential for effectiveness [20], the methods and principles of solution-focused brief therapy were chosen for the intervention. The emphasis of this approach is on encouraging clients/patients to set goals and achieve them by acknowledging and using their own strengths [21]. The positive, solution-focused philosophy – a coach-like way of working – is also relatively easy for health care professionals to adopt and implement [22,23].

At the beginning of the intervention in fall 2006, the participants in the intervention group received a personal, one-to-one lifestyle counseling session. In the session, the health nurse and the participant discussed the participant’s current health behaviors related to physical activity, dietary behaviors and/or sleep. The details of the discussion were determined by the participant’s needs, interests and the current life circumstances.

The aim was to keep the spirit of the discussion positive and encouraging: it started by focusing on the aspects of health behavior that the participant herself was pleased with, followed by additional positive feedback by the health nurse. If there were some aspects that the participant was not pleased with or she desired to change, she was encouraged to set a small behavioral goal. The health nurse then helped the participant in a coach-like manner to come up with the possible means to achieve the goal.

The health nurses recorded whether the participant chose to have this discussion. The topic (healthy physical activity and/or dietary behaviors and/or sleeping behaviors) and the duration of the discussion were also recorded. The participant was also offered printed material covering the topics discussed. Approximately 20 minutes were scheduled for each session.

Later, the girls in the intervention group took part in follow-up visits every six months for a total of 1.5–2.5 years, i.e. a total of 3 to 5 times, depending on how many follow-up visits the girl had left in the HPV-008 study routine at the time the LINDA intervention began. In these follow-up visits, the behavioral goals and the discussed themes were revisited briefly in a supportive manner. The topics of discussion in these visits were likewise determined by the girl herself. The intensity of the intervention was kept sufficiently low, and the content structured enough, to be implementable in primary care if it proved successful.

Prior to the beginning of the intervention, the health nurses received two four-hour group sessions for orientation and training in solution-focused philosophy and its implementation in the current study protocol. The main themes were *building collaboration* by adopting a non-authoritarian, supportive stance (e.g. listening and asking questions rather than offering advice) and *empowerment* by focusing on the positive (e.g. strengths and successes) and emphasizing freedom of choice. An additional document file describes the content of the training in more detail [see Additional file 1]. After the intervention had started, the nurses received approximately two hours of supervision in small groups, and an option to consult the psychologist responsible for their training and supervision, if required.

### Measures

The main outcomes of the present study were changes in the level of physical activity, meal regularity, bedtime before school- or work-days, and BMI. They were assessed by a self-report questionnaire on current health behaviors. BMI was assessed by anthropometric measures.

The girls in both groups completed a questionnaire at the beginning of the intervention and after the 1.5–2.5 year follow-up. At baseline, questions also asked about the participant’s current educational level, school success compared with classmates (based on the girls’ own judgment), and future educational plans. Questions assessing physical activity level, meal regularity and bedtime are described in Table 1. The questions were copied or adapted from previous self-report questionnaires that have been repeatedly used in nationwide health behavior surveys in Finland (e.g. *The School Health Promotion Study*, *the Adolescent Health and Lifestyle Survey*, *Health Behaviour and Health among the Finnish Adult Population*, and *The National FINRISK Study*).

**Table 1:** Description of questions assessing self-reported outcome variables

**Outcome variable**	**Description of assessment**
Physical activity level	“How much do you exercise and exert yourself physically during leisure time?
	If the amount varies a lot during the year, choose an option which best describes your AVERAGE situation.”
	Four response categories:
	1: During leisure time, I read, watch TV and do things where I don’t move much physically and which don’t exert me physically.
	2: During leisure time, I walk, ski (cross-country), bicycle, or move physically in some other way (e.g. do gardening) for at least four hours a week.
	3: During leisure time, I do dedicated fitness training for at least three hours a week.
	4: During leisure time, I regularly exercise competitively several times a week.
Meal regularity	“How often do you eat BREAKFAST (bread, cereals, porridge, yogurt, or something similar in the morning before going to school or work)?”
	“How often do you eat LUNCH (a warm meal at school, work or home or elsewhere around noon)?”
	“How often do you eat DINNER (a warm meal in the afternoon or the evening)?”
	Each has four response categories:
	1: every day;
	2: 4–6 times a week;
	3: 1–3 times a week;
	4: less than once a week.
Bedtime	“At what time do you usually go to sleep in the evenings preceding school-/work-days?”
	Response categories from 22:00 (or before) to 03:00 (or later) with a 30 min range of accuracy.

In the analyses, physical activity level was considered to have increased if the response to the question assessing physical activity level changed from physical inactivity (response category 1 in Table 1) to walking, actual fitness training or competitive athletics (response categories 2 to 4), or from walking (response category 2) to actual fitness training or competitive athletics (response categories 3 and 4). Changes in the opposite direction were considered as decreased physical activity. Actual fitness training and competitive athletics were both considered as a sufficient amount of healthy physical activity, and therefore changes between these two response categories were not assigned as increased or decreased physical activity. Likewise, meal (breakfast, lunch, dinner) regularities were considered more regular/irregular, and bedtime earlier/later according to the changes in the response categories of the respective questions in Table 1.

Height and weight were measured before and after the intervention unblinded by the same study nurse that had conducted the counseling discussion with the girl. Each vaccination center had similar height measuring instruments (Seca®), but the digital scales varied. Weight was also measured at each six-monthly revisit. The initial height was used for the BMI calculations before and after the intervention, as the growth in height of girls over 17 years can be considered to have ended [24]. The following BMI class division is used throughout the article: BMI < 18.5 for underweight, 18.5–24.9 for normal weight, 25.0–29.9 for overweight and > 30 for obese. Table 2 shows the distributions of all baseline characteristics in the intervention and control groups.

**Table 2:** Baseline characteristics in the intervention and control groups

**Characteristics**	**Intervention group (n = 1,349)**	**Control group (n = 1,338)**	**Difference between groups**
**Age (years)**			
Median (range)	19 (17–21)	19 (17–20)	p = 0.271^a^
**BMI**			
Median (interquartile range)	22.0 (4.0)	22.3 (4.1)	p = 0.029^a^
**BMI class (%)**			p = 0.470
<18.5	6.4	6.2	
18.5–24.9	73.8	71.8	
25.0–29.9	15.0	16.6	
>30	4.9	5.4	
**Physical activity level (%)**			p = 0.006
Inactive	25.5	29.5	
Walking, cycling etc. > 4 h/week	37.4	34.7	
Dedicated fitness training or competitive athletics > 3 h/week	37.1	35.8	
**Meal regularity (%)**			
Breakfast every day	57.1	55.9	p = 0.535
Lunch every day	46.8	47.4	p = 0.752
Dinner every day	29.8	32.5	p = 0.138
**Bedtime before school-/work-days (time)**			p = 0.760
≤22:00	13.4	14.0	
22:30	27.3	27.0	
23:00	30.0	29.5	
23:30	15.5	14.4	
≥24:00	13.8	15.1	
**Current education level (%)**			p = 0.004
High school	36.5	40.8	
Vocational school	26.3	26.9	
Combined high school + vocational school	5.5	7.7	
University	24.3	18.1	
Other	7.4	6.5	
**School success**^**b **^**(%)**			p = 0.125
Clearly above class average	10.2	8.9	
Slightly above class average	32.4	30.3	
Average	48.7	49.3	
Slightly below class average	7.7	10.2	
Clearly below class average	1.1	1.4	
**Future educational plans (%)**			p = 0.145
University	64.9	60.9	
Upper secondary school + vocational education	3.5	3.6	
Vocational education	9.2	8.8	
No studies	2.0	2.4	
Don’t know	20.3	24.3	

^a^Mann–Whitney test. Other p-values were achieved using Pearson’s Chi-square test.

^b^School success was based on the girls’ own judgments.

### Statistical analysis

The results were analyzed by the *intention-to-treat* (ITT) principle for those who had completed the questionnaire both at the baseline and at the end of the intervention period, and were not excluded for pregnancy during the intervention period. Of the 3,059 participants, 88% stayed in the intervention for the whole 1.5–2.5-year intervention period, and were included in the analysis.

The overall influence of the intervention was studied by the ITT principle by comparing the sum of changes in the three health behaviors under consideration: physical activity, meal regularity and bedtime before school- or work-days between the intervention and the control group. Thus, improvement in any of these health behaviors, with no decline in the other two behaviors, was interpreted as improved overall health behavior. This approach was selected because the goal of the intervention was to help the participant to enhance any of these health behaviors, not necessarily all of them, based on her needs and interests.

The analysis was also carried out by the *per-protocol* (PP) technique. That is, by analyzing the results for those participants in the intervention group with whom healthy physical activity and/or dietary behaviors and/or sleeping behaviors were actually discussed, based on the study records of the health nurse. The change in physical activity behaviors was analyzed for those who had a discussion (at least) on healthy physical activity behaviors. Similarly, the change in meal regularity was analyzed for those who had a discussion (at least) on healthy dietary behaviors, and bedtime for those who had a discussion (at least) on healthy sleeping behaviors. The change in BMI, on the other hand, was analyzed for those who had a discussion on physical activity, dietary behaviors, or sleeping behaviors, or any combination of them.

The effect of the intervention (in both the ITT and PP techniques) was analyzed by detecting the changes in the replies to the questions assessing the outcome variables (physical activity level, meal regularity and bedtime), or changes in BMI, before and after the intervention. The changes in these outcome variables were then compared between the groups.

The group median values of non-Gaussian distributed variables (e.g. individual BMI-changes) were tested with the Mann–Whitney test. The change in each categorical variable with ordinal scale (e.g. physical activity level) was first transformed into a new three-scale ordinal variable (change for better, no change, change for worse; as described above) and the difference in the occurrence of these new ordinal variables between the groups was tested using cross-tabulation and the Pearson’s Chi-squared test. The change in an ordinal variable within a group was tested using the McNemar-Bowker test, and the change in a group median value of a non-Gaussian distributed variable (e.g. BMI) within a group was tested using the Wilcoxon test. P-values of less than 0.05 were considered to be statistically significant. The statistical analyses were performed using IBM SPSS version 20. The number needed to treat (NNT) 95% confidence intervals were calculated using Confidence Interval Analysis software v.2.2.0 Build 57.

Cluster-randomized (or group-randomized) trials have a limited number of clusters, which makes it difficult for randomization to distribute potential sources of confounding evenly [25]. To account for this possible cluster effect, we first modified our ordinal outcome variables to be binary, and then used a mixed-model logistic regression analysis using community as a random effect and experimental condition (intervention vs. control group) as a fixed effect. This was done to verify that the results of our intervention remained the same after adjusting for the possible cluster effect, as was the case. The results are not shown. The cluster-effect analyses were carried out using the GLIMMIX procedure in SAS version 9.3.

### Ethical approval

The HPV-008 trial, including LINDA, has appropriate ethical approval from The National Advisory Board on Social Welfare and Health Care Ethics (ETENE).

## Results

In the intervention group, the median duration of the lifestyle counseling session was 15 minutes (IQR 10–17 min). Small – albeit statistically significant – differences between the intervention and control groups were found in baseline physical activity level, BMI and educational level (Table 2). There were fewer physically inactive participants (25% vs. 29%), BMI was slightly lower (22.0 kg/m^2^ vs. 22.3 kg/m^2^), and university-level education was more common (24% vs. 18%), in the intervention group compared with the control group.

### Intention-to-treat analysis

#### *Overall impact*

In the intervention group 37%, compared with 31% in the control group, improved at least one of the three target health behaviors by increasing their physical activity level, eating meals more regularly, or going to sleep earlier before school-/work-days, based on their needs and interests (Table 3), without a simultaneous decline in the other two target health behaviors. The number-needed-to-treat (NNT) was 18 (=1/(37.0%-31.4%)), 95% CI = 11–50. That is, for each 18-participant sub-group, one participant more in the intervention group than in the control group improved her health behaviors.

**Table 3:** **Changes in the outcome variables – intention-to-treat analysis**^**a**^

**Outcome variable**	**Intervention group (ITT) N = 1,349**	**Control group (CTRL) N = 1,338**	**Difference between groups (ITT vs. CTRL)**
	**%**	**%**	**p**^**b**^
**Overall impact**^**c **^**on the target health behaviors under consideration**	(n = 1,307)	(n = 1,292)	0.006
Improved	37.0	31.4	
No change	28.1	28.7	
Declined	34.9	39.9	
**Change in physical activity level**	(n = 1,330)	(n = 1,323)	0.771
Increased	23.8	23.1	
No change	58.8	58.5	
Decreased	17.4	18.4	
**Change in meal regularity**			
Breakfast	(n = 1,344)	(n = 1,332)	0.440
More regular	20.1	20.0	
No change	62.1	60.2	
More irregular	17.9	19.7	
Lunch	(n = 1,345)	(n = 1,336)	0.730
More regular	25.9	25.0	
No change	48.8	48.5	
More irregular	25.3	26.5	
Dinner	(n = 1,340)	(n = 1,334)	0.629
More regular	22.4	22.6	
No change	47.0	45.3	
More irregular	30.6	32.1	
**Change in the bedtime before school-/work-days**	(n = 1,343)	(n = 1,317)	0.061
Earlier	31.7	27.7	
No change	34.3	35.2	
Later	34.0	37.1	
	**Median (IQR)**	**Median (IQR)**	**p**^**d**^
**Change in BMI**	(n = 1,244)	(n = 1,294)	
All	0.55 (1.59)	0.51 (1.75)	0.769
BMI classes (before the intervention):			
BMI < 18.5	0.88 (1.21)	0.62 (1.30)	0.919
BMI 18.5–24.9	0.54 (1.55)	0.49 (1.57)	0.561
BMI 25.0–29.9	0.39 (2.08)	0.62 (2.55)	0.805
BMI ≥ 30	0.65 (3.01)	0.23 (3.80)	0.914

^a^All participants in the intervention and control arm who completed the questionnaire both at the baseline and at the end of the intervention period, and were not excluded for pregnancy during the intervention period.

^b^Pearson’s Chi-square test.

^c^Net effect (sum of changes) in the three health behaviors under choice: physical activity, meal regularity and bedtime before school- or work-days.

^d^Mann–Whitney test. CTRL = control, IQR = interquartile range.

#### *Physical activity*

During the intervention period, physical activity level improved in both groups (Table 4). In the intervention group, the number of physically inactive subjects decreased from 25% to 20%. The number of subjects who reported walking, cycling, cross-country skiing, or doing a similar activity at least for four hours per week increased slightly. Actual fitness training and/or competing athletics also increased slightly. In the control group, the change in the physical activity level was similar, and there was no statistically significant difference between the groups (Table 3).

**Table 4:** Distribution of the outcome variables before and after the intervention

	**Intervention group (ITT)**			**Control group**			**Intervention group (PP)**		
	**N = 1,349**			**N = 1,338**			**N**^**c**^		
**Outcome variable**	**Before (%)**	**After (%)**	**p**^**d**^	**Before (%)**	**After (%)**	**p**^**d**^	**Before (%)**	**After (%)**	**p**^**d**^
**Physical activity level**	(n = 1,330)		<0.001	(n = 1,323)		<0.001	(n = 629)		<0.001
Inactive	25.5	19.8		29.5	23.4		34.0	23.4	
Walking,cycling etc. > 4 h/wk	37.4	39.9		34.7	40.7		40.2	42.3	
Dedicat. fitness training or compet. athl. > 3 h/wk	37.1	40.2		35.8	35.8		25.8	34.3	
**Meal regularity**									
Breakfast	(n = 1,344)		0.501	(n = 1,332)		0.964	(n = 482)		0.222
Every day	57.1	58.9		55.9	55.4		52.7	55.8	
4–6 times/wk	16.4	16.1		17.9	17.9		18.5	18.9	
1–3 times/wk	15.4	14.8		14.7	15.3		16.2	14.5	
<1 time/wk	11.1	10.2		11.5	11.3		12.7	10.8	
Lunch	(n = 1,345)		0.254	(n = 1,336)		0.346	(n = 486)		0.254
Every day	46.8	45.9		47.4	46.6		40.5	44.2	
4–6 times/wk	35.9	36.1		34.2	34.3		37.9	35.2	
1–3 times/wk	12.8	14.1		14.6	13.6		15.8	16.7	
<1 time/wk	4.5	3.8		3.8	5.5		5.8	3.9	
Dinner	(n = 1,340)		0.006	(n = 1,334)		<0.001	(n = 484)		0.748
Every day	29.8	25.6		32.5	26.5		23.8	24.4	
4–6 times/wk	38.3	36.6		36.3	37.1		38.2	38.2	
1–3 times/wk	25.8	30.3		26.2	29.7		30.6	28.5	
<1 time/wk	6.1	7.5		5.0	6.7		7.4	8.9	
**Bedtime before school/workdays**	(n = 1,343)		0.049	(n = 1,317)		<0.001	(n = 266)		0.318
≤22:00	13.4	13.5		14.0	12.6		7.1	10.5	
22:30	27.3	26.3		27.0	22.6		22.6	20.7	
23:00	30.0	30.9		29.5	31.8		25.6	28.2	
23:30	15.5	13.0		14.4	14.0		22.9	22.2	
≥24:00	13.8	16.4		15.1	19.0		21.8	18.4	
**BMI class**	(n = 1,243)		<0.001	(n = 1,293)		<0.001	(n = 918)		<0.001
< 18.5	6.4	4.3		6.2	3.8		5.2	4.1	
18.5–24.9	73.8	70.0		71.8	68.2		73.3	69.2	
25.0–29.9	15.0	19.7		16.6	20.2		15.4	19.4	
≥ 30	4.9	6.0		5.4	7.8		6.1	7.3	

The outcome variables are analyzed in the intervention group (ITT) according to the intention-to-treat principle^a^, and in the intervention group (PP) according to the per-protocol principle^b^.

^a^All participants in the intervention arm who completed the questionnaire both at the baseline and at the end of the intervention period, and were not excluded for pregnancy during the intervention period.

^b^The participants in the intervention arm who completed the questionnaire both at the baseline and at the end of the intervention period, were not excluded for pregnancy during the intervention period, AND chose to discuss healthy physical activity and/or dietary behaviors and/or sleeping behaviors with the study nurses.

^c^The size of the intervention group in the per-protocol analyses varied according to the outcome variable as follows: Regarding change in physical activity level, participants who chose to discuss (at least) their physical activity behaviors (n = 637) were included. A valid answer to the question assessing physical activity was given by n = 629. Regarding change in meal regularity, participants who chose to discuss (at least) their dietary behaviors (n = 486) were included. Regarding change in bedtime, participants who chose to discuss (at least) their sleeping behaviors (n = 269) were included. Regarding change in BMI, participants who chose to discuss either their physical activity or dietary behaviors or sleeping behaviors, or two or three of these (n = 957) were included.

^d^McNemar-Bowker test for testing the within-group change.

#### *Regularity of meals*

Concerning dinner, there was a statistically significant tendency towards greater irregularity in both groups (Table 4), but no significant difference between the groups (Table 3). Breakfast and lunch regularity remained materially unchanged in both groups (Table 4).

#### *Bedtime before school- or work-days*

In both groups, there was a small – albeit statistically significant – tendency towards later bedtime before school- or work-days after the intervention, but no statistically significant difference between the groups was observed (Tables 3 and 4). For example, the percentage of those participants who went to sleep after midnight increased from 14% to 16% in the intervention group vs. from 15% to 19% in the control group (Table 4). In the intervention group, 32% went to sleep earlier and 34% later than before the intervention, compared with 28% and 37% in the control group (Table 3).

#### *Body mass index*

Individual BMI values increased in both groups (Wilcoxon test p < 0.001). The group median values of the individual BMI changes were +0.55 kg/m^2^ in the intervention group and +0.51 kg/m^2^ in the control group (Table 3). These correspond to median weight gains of 1.5 kg and 1.4 kg, respectively. The percentage of overweight participants increased in the intervention group from 15% to 20% vs. 17% to 20% in the control group, and the percentage of obese participants from 5% to 6% vs. 5% to 8%, respectively (Table 4). The BMI changes were not different between the groups (Table 3).

### Per-protocol analysis

#### *Physical activity*

In the intervention group, 637 (47%) participants chose to discuss their physical activity behaviors and the possibilities of improving them. Of them, 30%, compared with 23% in the control group, succeeded in increasing their physical activity level (Table 5). The number needed to treat was 15, 95% CI = 9 ─ 38. That is, for every intervention conversation held with 15 participants, one participant more in the intervention group than in the control group improved her physical activity over time. The number of physically inactive participants of the intervention group decreased from 34% to 23% (Table 4), compared with 29% to 23% in the control group.

**Table 5:** **Changes in outcome variables ─ per-protocol analysis**^**a**^

**Outcome variable**	**Intervention group (PP) N**^**b**^	**Control group (CTRL) N = 1,338**	**Difference between groups (PP vs. CTRL)**
	**%**	**%**	**p**^**c**^
**Change in physical activity level**	(n = 629)	(n = 1,323)	0.003
Increased	30.0	23.1	
No change	54.8	58.5	
Decreased	15.1	18.4	
**Change in meal regularity**			
Breakfast	(n = 482)	(n = 1,332)	0.491
More regular	22.6	20.0	
No change	58.3	60.2	
More irregular	19.1	19.7	
Lunch	(n = 486)	(n = 1,336)	0.110
More regular	29.8	25.0	
No change	46.1	48.5	
More irregular	24.1	26.5	
Dinner	(n = 484)	(n = 1,334)	0.024
More regular	24.6	22.6	
No change	50.0	45.3	
More irregular	25.4	32.1	
**Change in the bedtime before school-/work-days**	(n = 266)	(n = 1,317)	0.016
Earlier	35.7	27.7	
No change	34.6	35.2	
Later	29.7	37.1	
	**Median (IQR)**	**Median (IQR)**	**p**^**d**^
**Change in BMI**	(n = 918)	(n = 1,294)	
All	0.52 (1.53)	0.51 (1.75)	0.996
BMI classes (before the intervention):			
BMI < 18.5	0.71 (1.08)	0.62 (1.30)	0.495
BMI 18.5–24.9	0.51 (1.48)	0.49 (1.57)	0.740
BMI 25.0–29.9	0.45 (2.28)	0.62 (2.55)	0.929
BMI ≥ 30	0.57 (2.94)	0.23 (3.80)	0.881

^a^The participants in the intervention arm who completed the questionnaire both at the baseline and at the end of the intervention period, were not excluded for pregnancy during the intervention period, AND chose to discuss healthy physical activity and/or dietary behaviors and/or sleeping behaviors with the study nurses.

^b^The size of the intervention group in the per-protocol analyses varied according to the outcome variable as follows: Regarding change in physical activity level, participants who chose to discuss (at least) their physical activity behaviors (n = 637) were included. A valid answer to the question assessing physical activity was given by n = 629. Regarding change in meal regularity, participants who chose to discuss (at least) their dietary behaviors (n = 486) were included. Regarding change in bedtime, participants who chose to discuss (at least) their sleeping behaviors (n = 269) were included. Regarding change in BMI, participants who chose to discuss either their physical activity or dietary behaviors or sleeping behaviors, or two or three of these (n = 957) were included.

^c^Pearson’s Chi-square test.

^d^Mann–Whitney test.

PP = per-protocol, CTRL = control, IQR = interquartile range.

#### *Regularity of meals*

In the intervention group, 486 (36%) participants chose to discuss their dietary behaviors and the possibilities of improving them. Dinner irregularity also increased among these participants, but less than among the control group (Table 5). The number needed to treat was 15, 95% CI = 9–46.

#### *Bedtime before school- or work-days*

In the intervention group, 269 (20%) participants chose to discuss their sleeping behaviors and the possibilities of improving them. Of these participants, 36% went to sleep earlier and 30% later than before the intervention, compared with 28% and 37% in the control group, respectively (Table 5). The number needed to treat was 13, 95% CI = 7–61.

#### *Body mass index*

In the intervention group, 957 (71%) participants chose to discuss any of the target health behaviors: their physical activity and/or dietary behaviors and/or sleeping behaviors, and the possibilities of improving them. In the per-protocol analyses, the changes of BMIs in the intervention and control groups did not differ (Table 5).

## Discussion

The LINDA lifestyle intervention consisted of individualized, solution-focused low-intensity intervention with long-term support and motivation for promoting healthy dietary and sleeping behaviors and physical activity. The intervention had a small positive overall (net) effect on the target health behaviors of all young women who participated in the intervention, as compared with the controls. In addition, it was beneficial regarding physical activity level, dinner regularity and bedtime before school-/work-days among those who actually had the counseling discussion with the trained health nurses, as compared with the controls. In particular, the proportion of physically inactive girls decreased from 34% to 23%, versus from 29% to 23% in the control group, and most importantly, the intervention had no harmful effects on any of the study outcome variables.

The effectiveness of lifestyle interventions in helping people to achieve dietary change or weight loss seems to depend on the intensity of the intervention [20]. Our study had a lower intensity than the previous lifestyle interventions that most closely resembled our intervention [14-17]. Our study also had a lower – but still very similar – effectiveness than the PACE + study [14]. In that study [14], the positive changes in the control group were considered as a measurement reactivity phenomenon: self-reported behavior is influenced by the measurement process itself, i.e., repeated assessments and surveys on health behaviors may also motivate the control group participants to change behavior. This may also well be the reason for the positive changes in the control group in our study.

Consistent with the previous studies [14-17], our study suggests that changes in physical activity are more easily achieved compared with changes in diet. It seems that even low intensity individual counseling, such as ours, is enough to yield long-term positive changes in physical activity. This is in line with the recent review of reviews [20] where the authors found no relationship between lifestyle intervention intensity and physical activity outcomes in adults. Finally, our results suggest that the solution-focused principles and methods are usable in the promotion of healthier behaviors, and that this coach-like, collaborative, empowering and positive approach may be taught relatively quickly to those implementing the intervention.

The major strengths of the present study include the large sample size, a long maintenance period, and individually tailored personally relevant intervention content. This combination has been missing from the previous lifestyle intervention studies with young adults [14-17,26,27], and has been called for in the latest review articles [26,27]. In addition, adherence to the intervention was high (88%).

There were also some limitations to our study. After the cluster-randomization, there were still some baseline differences in the level of physical activity, BMI, and the current educational level between the intervention and control groups. According to Greaves et al. [20] four reviews disagreed as to whether the targeting of interventions at people who are more sedentary was associated with greater increases in the amount of physical activity. The same was true for baseline BMI [20]. In another study, one also conducted in Finland, socioeconomic position (impact of the level of education and occupation) did not seem to have any impact on the effectiveness of lifestyle intervention in individuals at high risk for type 2 diabetes [28]. Therefore, we have no particular reason to suspect strong biases in these respects in either direction.

Another limitation is the limited set of questions in the questionnaire for detecting the change in dietary behaviors. The intervention may have resulted in changes towards healthier dietary behaviors, e.g. more vegetables and less candy, which were not detected because they did not affect the meal regularity. Furthermore, the data are self-reported and the answers about health behaviors may depend on the expected social desirability of these behaviors among those who performed the intervention [29]. However, there is evidence that reliability and validity of self-reported behaviors among adolescents is good [30-32]. Concerning the question in our study assessing the physical activity level, a moderate correlation has been found between the response to this question and the accelerometer measurements, with the exception of the sedentary variable [33]. In addition, survey-reported school-night bedtimes have been shown to correspond well to those reported by diary and estimated with actigraphy [34]. Even if there were any bias in reporting, it is assumed to be the same for both groups (intervention and control), so the comparison is still valid. Likewise, any variation in the repeatability of the answers adds noise, but should not bias the group comparisons. Lastly, in line with the above-mentioned measurement reactivity phenomenon [14], unintentional spreading of the intervention principles from the intervention study nurses to their colleagues in the control group (“contamination”) could have also led to positive changes in the control group, thus masking the effects of the intervention in the comparison.

Generalizing the results of this intervention into common clinical practice is not straightforward. Our study population consisted of young healthy females who may have had more interest in health-related issues compared with the overall population, as they accepted the HPV vaccination study invitation (20% of invitees) and the LINDA invitation (87% of the invitees). This could overestimate the positive result. On the other hand, in student health care, most visitors are healthy young people (attending e.g. for contraceptive pills), which is why this method may work well with them. Currently, healthy young adults do not have the opportunity for lifestyle counseling and weight monitoring in primary care every six months like the control group did in our study. Thus, the impact of the intervention compared with standard care may be actually higher than estimated in our study. Furthermore, the participants at the baseline of the present study did not have a long-term relationship with the study health nurses beforehand, which could undermine the impact of the intervention. A trusting relationship with good communication between patient and health care provider, known to be beneficial for health outcomes [35], often takes time to build.

The role of motivation is central in behavior change, and motivation is the key element of behavior interventions. Motivation is a continuum, not an on-off phenomenon [36]. It is not inherent within the participant, rather, it stems from the *interaction* between the participant/patient and the health care provider [37]. Therefore, the beneficial intervention effects of the present study may reflect both the interests of the participants and the motivating skills of the study nurses trained in solution-focused discussions. Factors explaining the successful facilitation of health behavior change remain to be examined in future studies.

### Implications

The low-intensity primary intervention was effective in helping young women to make a beneficial long-term net improvement in their health behaviors concerning physical activity, meal regularity and/or bedtime before school-/work-days. The participant’s needs and interests determined the discussed topic(s) and change target(s), if any. If a participant did not choose the counseling in our study, no resources (time from the health nurses) were expended. The number needed to treat (NNT) to achieve such a long-term net improvement as compared with standard care was 18, (95% CI = 11 ─ 50). Realizing how difficult a long-term lifestyle change is, and how much it benefits the patient in long-term, the NNT seems sufficiently low, and the low-intensity-intervention seems worth the effort for health care practitioners.

The proportion of physically inactive participants was higher among those who were willing to discuss these health behaviors compared with the control group. After the intervention, the proportion of physically inactive participants had decreased to the same level as the control group, which means that the intervention was able to reach those who especially needed the positive change the most. A similar change happened with bedtimes.

Finally, it should be noted that while the BMI increased overall in all BMI classes in both groups during the intervention period, around 10% of the participants with initially normal weight became overweight. Thus, if the proposed intervention actions were insufficient to prevent adverse weight gain among adolescents becoming young women, the improvement in the physical activity level is of great importance, as physical activity also seems to have beneficial effects on health independent of weight [38].

Future studies are needed on the details of why some participants succeed in health behavior change and maintenance, and how this could be supported in public health care to yield higher success rates.

## Conclusions

The solution-focused brief therapy intervention helped to make a small, long-term overall improvement in the health behaviors of physical activity, meal regularity and/or earlier bedtime among young women. The participants were allowed to choose the discussed health behavior according to their interests, if they had any. Physical activity and earlier bedtime in particular can be positively influenced by a solution-focused low-intensity intervention.

## Abbreviations

BMI: Body mass index; HPV: Human papilloma virus; NNT: Number needed to treat.

## Competing interests

The authors declare that they have no competing interests.

## Authors’ contributions

PV carried out the planning of the present study, performed the statistical analysis and drafted the manuscript. SL-J participated in the design of the LINDA study and the planning of the present study and helped to draft the manuscript. SA and AR participated in the conception and design of the LINDA study, SA bore the main responsibility for planning the content of the intervention, training the study nurses, and acquisition of data, and participated in the planning of the present study and helped to draft the manuscript. M-TS and PL contributed to the conception and design of the LINDA study, and TE contributed to organizing recruitment of study participants and the data collection of this study. ML contributed to the conception, design and acquisition of the data of the LINDA study, and helped in revising the manuscript. All authors read and approved the final manuscript.

## Pre-publication history

The pre-publication history for this paper can be accessed here:

http://www.biomedcentral.com/1471-2458/13/1044/prepub

## Supplementary Material

Additional file 1 A brief manual for the LINDA intervention. Click here for file
