# Supplementary material for: LINDA – a solution-focused low-intensity intervention aimed at improving health behaviors of young females: a cluster-randomized controlled trial
Source: BMC Public Health. 2013 Nov 4;13:1044. doi: 10.1186/1471-2458-13-1044 (PMC4228239; doi:10.1186/1471-2458-13-1044)
Supplement: Additional file 1 — A brief manual for the LINDA intervention. [file 1471-2458-13-1044-S1.pdf]

A Brief Manual for the LINDA Intervention

<u>Aims</u>

Primary aim: The promotion of the participants' well-being and healthy habits.
Secondary aim: The promotion of the participants' long-term weight management.

<u>Main principle</u>

Good interaction skills and client-centeredness are at the core of motivating and influencing others. Offering advice is often interpreted as authoritarian, and it rarely works in lifestyle counseling.

<u>Methods of promoting cooperation and change</u>

Principles and techniques of solution-focused brief therapy are used to *empower* clients. Empowered clients feel willing and able to take responsibility for their well-being and health.

In a *solution-focused* way of working, the professional focuses on the client's hopes for the future, her personal strengths, and what is already working well for her. Client successes, rather than her problems and their causes, are explored.

<u>Suggested techniques</u>

Avoid "knowing better". Instead, highlight client autonomy and trust in her competence by *listening, asking (open) questions, and creating freedom of choice.*

Course of the first intervention discussion

1) Start with client's own thoughts on her current health habits. What is she pleased (or displeased) with, and why?
2) Focus on everything that is working well.
3) Prompt the client to consider setting a goal to change some behavior that she is not pleased with. Help her form a goal that is sufficiently realistic, practical and specific.
4) Discuss the advantages of achieving the goal. Start with the client's own views.
5) Ask about resources, personal strengths, and client's successes. Look for them throughout the discussion. Create ideas together on how the client could use her resources in behavior change.

Course of the follow-up discussions

1) Again, start with client's own thoughts on her health habits and the progression toward achieving the goal. What is she pleased with, what is better?
2) Look for and focus on every positive change in health behavior, and every step taken towards the goal, no matter how small.
3) Give positive feedback for good changes, and even for maintaining what was working well before. Encourage the client to keep up the good work.
4) Remind the client of her abilities. What made the good changes possible?

5) If the client has achieved the goal, would she be willing to set another small goal? If the client is displeased with her poor progress, does she want to change her goal?
